# Chlorogenic Acid Prevents UVA-Induced Skin Photoaging through Regulating Collagen Metabolism and Apoptosis in Human Dermal Fibroblasts

**DOI:** 10.3390/ijms23136941

**Published:** 2022-06-22

**Authors:** Nina Xue, Ying Liu, Jing Jin, Ming Ji, Xiaoguang Chen

**Affiliations:** State Key Laboratory of Bioactive Substance and Function of Natural Medicines, Institute of Materia Medica, Chinese Academy of Medical Sciences and Peking Union Medical College, Beijing 100050, China; angelnina@imm.ac.cn (N.X.); liuying@imm.ac.cn (Y.L.); rebeccagold@imm.ac.cn (J.J.); jiming@imm.ac.cn (M.J.)

**Keywords:** collagen, matrix metalloproteinases, human dermal fibroblasts, photoaging, chlorogenic acid, UVA radiation

## Abstract

Skin aging is categorized as chronological aging and photo-aging that affected by intrinsic and extrinsic factors. The present study aimed to investigate the anti-aging ability and its underlying mechanism of chlorogenic acid (CGA) on human dermal fibroblasts (HDFs). In this study, CGA specifically up-regulated collagen I (Col1) mRNA and protein expressions and increased the collagen secretion in the supernatant of HDFs without affecting the cell viability, the latter was also demonstrated in BioMAP HDF3CGF system. Under ultraviolet A (UVA)-induced photoaging, CGA regulated collagen metabolism by increasing Col1 expression and decreasing matrix metalloproteinase 1 (MMP1) and MMP3 levels in UVA-irradiated HDFs. The activation of transforming growth factor-β (TGF-β)-mediated Smad2/3 molecules, which is crucial in Col1 synthesis, was suppressed by UVA irradiation and but enhanced at the presence of CGA. In addition, CGA reduced the accumulation of UVA-induced reactive oxygen species (ROS), attenuated the DNA damage and promoted cell repair, resulting in reducing the apoptosis of UVA-irradiated HDFs. In conclusion, our study, for the first time, demonstrate that CGA has protective effects during skin photoaging, especially triggered by UVA-irradiation, and provide rationales for further investigation of CGA being used to prevent or treat skin aging.

## 1. Introduction

Aging is a continuous and slow process that compromise the morphological and functional characteristics of different organs and systems in humans and in animals [1]. Skin aging is only one visible part of this process, which is characterized by appearance of wrinkles, laxity, and pigmentary irregularities induced by intrinsic (e.g., chronological, hormonal, and genetic) and extrinsic environmental stress factors. These histological changes are typically related to keratinocyte and melanocyte hyperproliferation and degradation of collagen fibers in dermis [2,3].

The skin consists of an outer epidermal layer and an inner dermal layer which are connected by the basement membrane. Type I collagen (Col1), the most abundant dermal extracellular matrix (ECM) component, associates with collagen III (Col3) to form a broad extracellular fiber in the dermis of adult skin. Col1 accounts for 85–90% while Col3 is 8–11% in human adult skin, the latter is a more important component in neonatal skin [4]. Col1 is decreased with chronological-aging, but can be triggered by photo-aging [5]. In addition, Col1 can be degraded by hydrolyzing proteins such as matrix metalloproteinases (MMPs), which are a family of ubiquitous endopeptidases that degrade ECM proteins [6]. Previous studies have demonstrated that MMP-1, MMP-2, and MMP-3 are mainly MMPs for degrading collagens and elastin [7]. With the aging process of the skin, the collagen biosynthesis decreases, and collagen fragmentation increases, which both contribute to an overall reduction in collagen content [8,9,10]. So, in recent years, many nutritional supplements and specialty foods containing collagen have been marketed to facilitate anti-aging of the skin.

Natural products or molecules have been long to be used to improve the appearance of the skin, such as arbutin, ramulus mori extract, azelaic acid, β-hydroxy acids, lactic acid, chamomile extract, and ellagic acid [11]. Many of these natural small molecules or extracts exhibit antioxidant activity and play roles in maintaining homeostasis and preventing photo-aging due to ultraviolet (UV)-induced skin damage. While this may serve as a general mechanism, more defined molecular mediators are difficult to identify due to the mixed components in many natural products. Chlorogenic acid (CGA), an ester of L-quinic acid and caffeic acid, is a natural product commonly found in the human diet and in beverages prepared from herbs, fruits, and vegetables [12]. It has been shown to have several physiological properties, including anti-inflammatory, anti-oxidant, immune modulation, anti-bacterial and anti-tumor effects [13,14,15,16]. There have been a few studies that reported the anti-aging effect of CGA. For example, Li et al. reported that CGA may suppress melanogenesis in B16 melanoma cells by inhibition of tyrosinase activity, implying the role of CGA on skin pigmentation [17]. Cha and Her et al. showed that CGA could prevent against the skin aging induced by UVB radiation [18,19,20]. These reports were related to the protective effect of CGA against UVB-induced photoaging in keratinocytes, fibroblasts derived from newborn foreskin or hairless mice, as extrinsic skin aging is mainly associated with the solar ultraviolet (UV) radiation exposure [21]. However, UVB mainly leads to the damage of epidermal layer. Approximately 90–99% of the longwave ultraviolet rays (UVA) in sunlight penetrate more deeply into the human skin dermal tissue, thus UVA is a primary factor for skin photoaging [22].

In that regard, there have not been studies to show if and how CGA protects human dermal fibroblast adult (HDF-α) cells under normal and UVA-induced photoaging conditions. In this study, we focused on the antiphotoaging effect of CGA on HDFs, particularly under UVA-irradiated stress condition and the underlying mechanism associated with collagen metabolism and apoptosis.

## 2. Results

### 2.1. Effect of CGA on Collagen Expression in Human Skin Fibroblasts

**Figure 1:**
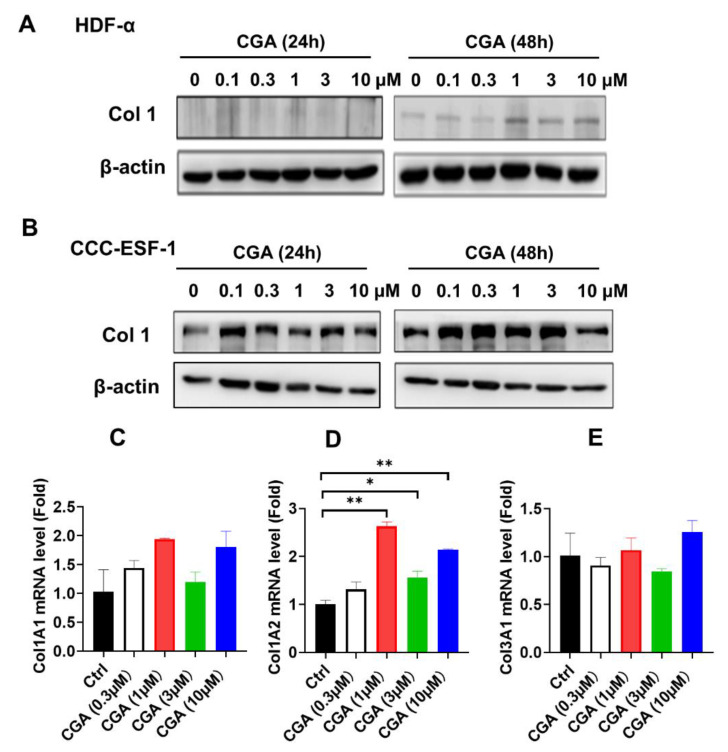
The effect of CGA on Collagen expression in human skin fibroblasts. The collagen I levels were detected by western blot assay after exposure of CGA for 24 or 48 h in HDF-α cells (**A**) and in CCC-ESF-1 cells (**B**). The Col1A1 (**C**), Col1A2 (**D**) and Col3A1 (**E**) mRNA levels were measured by qRT-PCR assay after treatment with CGA for 48 h in HDF-α cells. The values are expressed as the mean ± SD from three experiments. * *p* ≤ 0.05, ** *p* ≤ 0.01 indicate a significant difference.

The effect of CGA on the collagen expression in human skin fibroblasts were first measured by western blot and quantitative RT-PCR (qRT-PCR) assay. As shown in Figure 1A,B, CGA used at lower concentration from 0.1 to 3 μM increased the type 1 collagen (Col1) protein expressions in both HDF-α and CCC-ESF-1 cell. And the stimulatory effect on Col1 protein expressions was more obvious at exposure of CGA for 48 h. To examine whether the stimulatory effect of CGA on collagen occurred at the transcriptional level, the genes of Col1 (Col1A1, Col1A2) and Col3 (Col3A1) mRNA expressions were detected. As shown in Figure 1C–E, CGA at concentration of 0.3 to 10 μM enhanced the Col1A1 and Col1A2 mRNA levels, especially in Col1A2 mRNA expression. Presence of CGA (1, 3 and 10 μM) significantly increased the Col1A2 mRNA levels. However, the Col3A1 mRNA levels were almost unchanged after exposure of CGA. These data suggest that CGA specifically induced the Col1 expressions; this result was attributed to the promotion of Col1A2 gene transcription.

### 2.2. Effect of CGA on Collagen Secretion in HDFs

**Figure 2:**
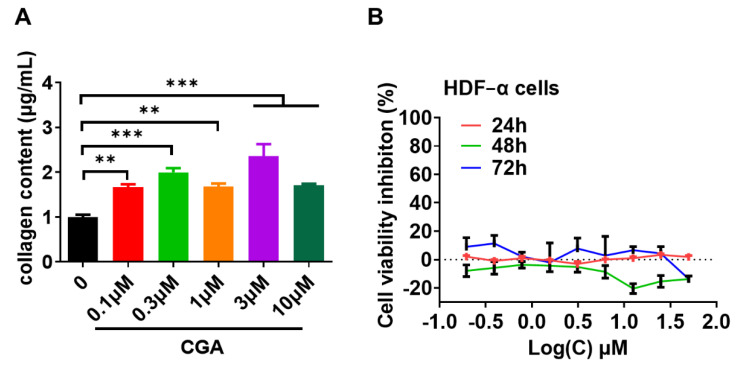
The effect of CGA on collagen secretion in HDF-α cells. (**A**) The soluble total collagen content in the supernatant of HDF-α cells were measured after exposure of CGA for 48 h. (**B**) The cell viability inhibition of HDF-α cells treated with CGA for 24, 48, or 72 h were detected by MTT assay. The values are expressed as the mean ± SD. from three experiments. ** *p* ≤ 0.01, *** *p* ≤ 0.001 indicate a significant difference.

**Figure 3:**
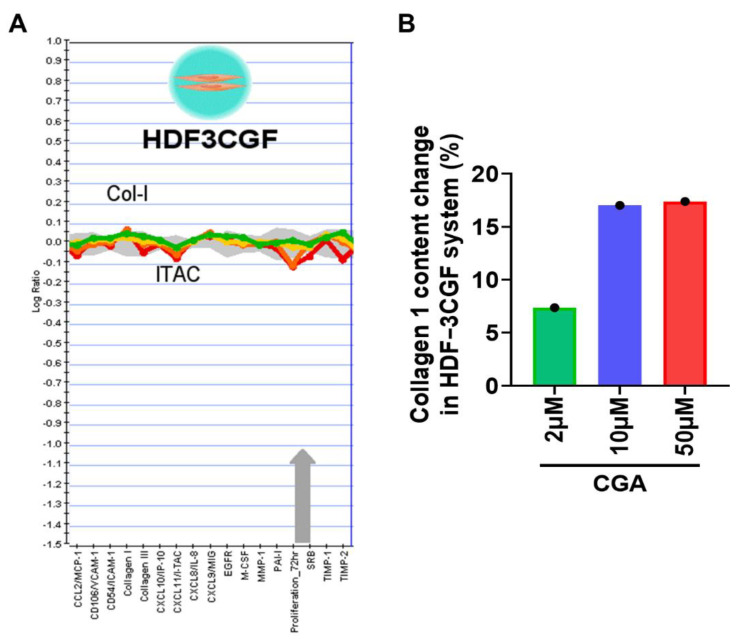
The effect of CGA on collagen I productions in HDFs. (**A**) The BioMAP HDF3CGF system was used to evaluate the effect of CGA on 16 biomarkers (proteins) involved in the tissue remolding and inflammation. (**B**) The relative increased percentages of collagen I after treatment with CGA were quantified and shown in the histogram.

We next focused on HDF-α cells, which were exposed to indicated doses of CGA for 48 h and the secreted amount of total collagen in the cell culture supernatant was assessed by Biocolor Sircol^TM^ assay. Exposure to CGA resulted in a significant induction of collagen secretion (Figure 2A). Meanwhile, the effect of CGA on cell viability were detected by MTT assay. Figure 2B showed that CGA doses between 0.3 to 50 μM almost did not affect the proliferation of HDF-α cells after treatment for 24, 48, and 72 h. The results implying that CGA promoted the collagen production without affecting the proliferation of HDF-α cells. In addition, in the BioMAP HDF3CGF system, primary human neonatal foreskin fibroblasts (HDF-n) were plated in low serum conditions 24 h before stimulation with cytokines listed in Table 1 and treated with indicated doses of CGA for additional 72 h. Sixteen (16) parameters described in Table 1 were evaluated for the effect of CGA on HDFs. Among these readouts, the tissue-remodeling molecule collagen I and inflammatory factor interferon-inducible T-cell alpha chemoattractant (I-TAC, also known as CXCL11) changed most obviously after treatment with CGA in this system (Figure 3A). CGA at the concentration of 10 and 50 μM increased the collagen I content by approximately 17% (Figure 3B).

### 2.3. Effect of CGA on Collagen Metabolism in UVA-Irradiated HDFs

**Figure 4:**
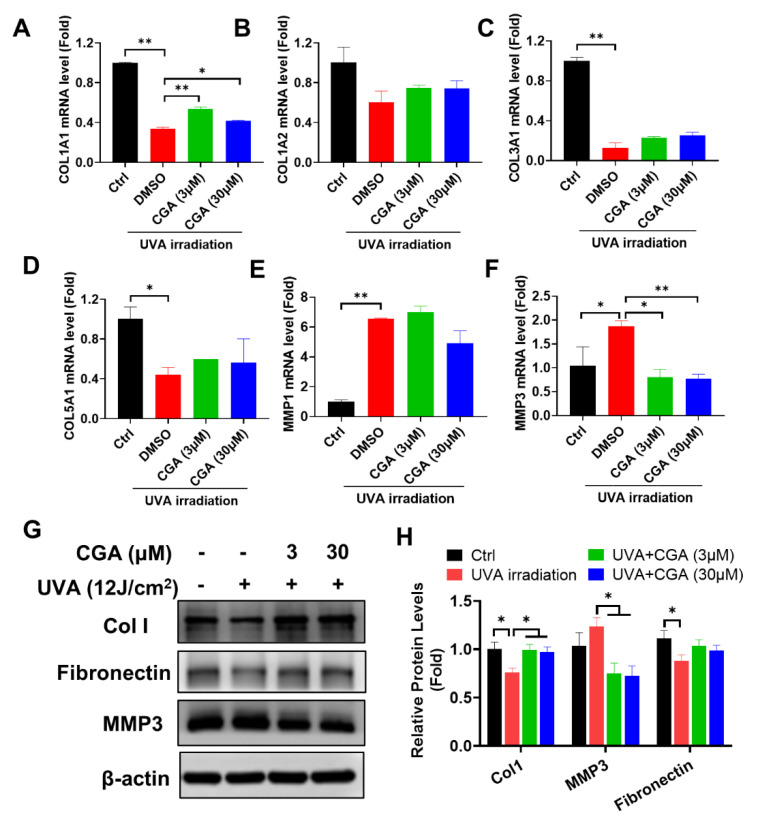
The effect of CGA on collagen I metabolism in UVA-irradiated HDF-α cells. The mRNA levels of Col1A1 (**A**), Col1A2 (**B**), Col3A1 (**C**), Col5A1 (**D**), MMP1 (**E**) and MMP3 (**F**) in UVA-irradiated HDF-α cells were detected by qRT-PCR. Data represent the mean ± SD of three independent measurements. * *p* ≤ 0.05, ** *p* ≤ 0.01 indicate a significant difference. (**G**) The Col1, MMP3 and fibronectin protein expressions in UVA-irradiated HDF-α cells were measured by western blot assay. (**H**) Relative protein expressions were quantified by densitometry and shown in the histogram. The results represent the averages of three independent experiments. * *p* ≤ 0.05 indicate a significant difference.

Skin photoaging is typically associated with UV irradiation. Based on our study on HDFs under non-stressed condition, we further investigated CGA’s role for skin photoaging triggered by UVA irradiation. Particularly, we measured the collagen metabolism from its transcriptional synthesis and degradation. Firstly, we detected the mRNA levels of key genes involved in different subsets of collagen synthesis in UVA-irradiated HDF-α cells. Figure 4A–D showed that type I collagen (Col1A1), type III collagen (Col3A1) and type V collagen (Col5A1) gene levels were all significantly down-regulated after UVA irradiation. At the presence of CGA (3 and 30 μM), Col 1A1 gene expression was remarkably increased in UVA-irradiated HDF-α cells (*p* ≤ 0.01; *p* ≤ 0.05), but CGA had minimal effects on Col3A1 or Col5A1 mRNA levels. We also investigated the effects of CGA on the MMPs expressions, such as MMP1, MMP3, and MMP9, which were involved in the breakdown of collagen. As opposite to collagens, UVA-irradiation significantly increased the mRNA expressions of MMP1 and MMP3 (*p* ≤ 0.01; *p* ≤ 0.05; Figure 4E,F). However, the combined treatment with CGA plus UVA irradiation led to the decrease of MMP1 and MMP3 transcriptional expression in HDF-α cells, compared with the UVA irradiation alone. And the MMP3 mRNA levels were significantly decreased by 3 and 30 μM CGA in UVA-irradiated HDF-α cells (*p* ≤ 0.01; *p* ≤ 0.01).

In addition, we analyzed the effect of CGA on the protein levels of Col1 and MMP3 (Figure 4G,H). Consistent with mRNA data, UVA irradiation significantly reduced the Col1 protein, but the reduction was rescued by CGA treatment in HDF-α cells. In contrast, MMP3 protein was slightly increased after UV irradiation, but downregulated by combination with CGA. The level of fibronectin, another ECM protein, showed the similar trend as Col1 after UVA-irradiation or in presence of CGA in HDF-α cells.

### 2.4. Effect of CGA on TGF-β/Smad Signaling in UVA-Irradiated HDFs

**Figure 5:**
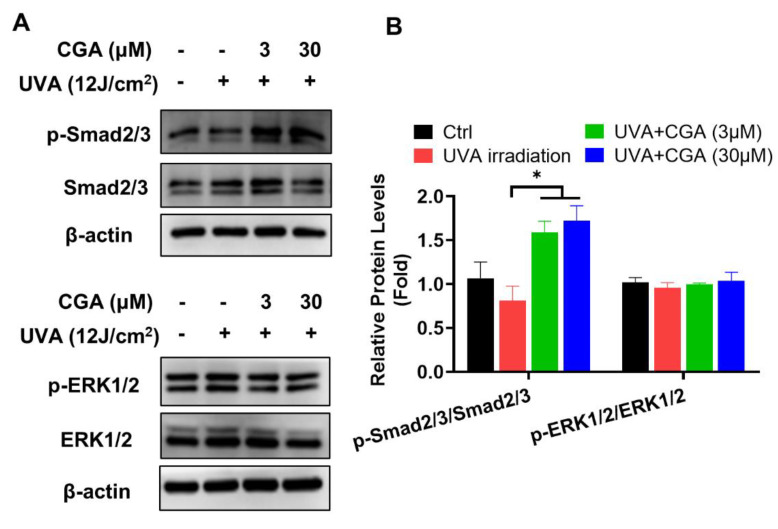
The effect of CGA on Smad2/3 and ERK1/2 signaling pathways in UVA-irradiated HDF-α cells. (**A**) The p-Smad2/3, Smad2/3, p-ERK1/2 and ERK1/2 protein expressions in UVA-irradiated HDF-α cells were measured by western blot assay. (**B**) Relative protein expressions were quantified by densitometry and shown in the histogram. Data are expressed as the mean from three experiments. * *p* ≤ 0.05 indicate a significant difference.

To explore the underlying mechanism of CGA-induced collagen production in UVA-irradiated HDFs, the activation of Smad2/3 and ERK1/2 molecules, two crucial signaling pathways of collagen synthesis [23,24], were analyzed by Western blotting. After exposure to UVA radiation, the level of Smad2/3 phosphorylation were decreased in HDF-α cells. Combination treatment with UVA irradiation and CGA increased the phosphorylated Smad2/3 expressions compared to those of only UVA-irradiated HDF-α cells. However, the levels of phosphorylated ERK1/2 were unchanged after exposure to UVA or the combination with CGA (Figure 5A,B).

### 2.5. CGA Protects against the UVA-Induced Apoptosis in HDFs

**Figure 6:**
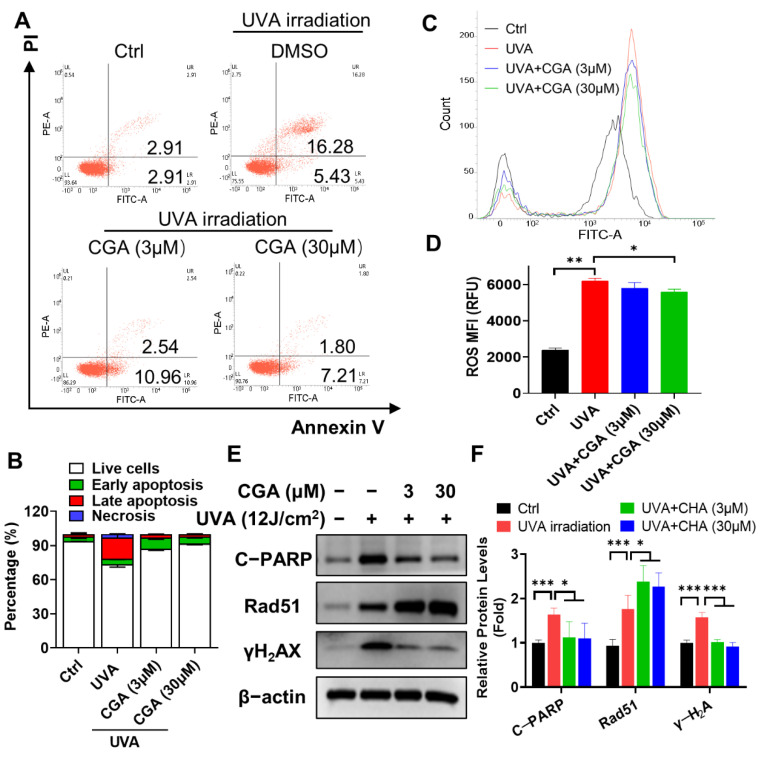
The effect of CGA on cell apoptosis and ROS production in UVA-irradiated HDF-α cells. HDF-α cells were irradiated by UVA for 60 min, followed by treatment with CGA for 24 h, and harvested for apoptosis and ROS analysis. (**A**) The percentage of cell apoptosis were determined by an annexin V-FITC/PI double staining assay followed by flow cytometry. (**B**) Statistical analysis of the live cells, early and late apoptotic cells and necrosis cells in various treatment groups. Data are expressed as the mean from three experiments. (**C**) ROS production was evaluated using DCFH-DA probe by flow cytometry assay. (**D**) The ROS mean fluorescent intensity (MFI) were calculated from three experiments and shown in the histogram. * *p* ≤ 0.05, ** *p* ≤ 0.01 indicate a significant difference. (**E**) The protein expressions of cleaved PARP, γ-H_2_AX and Rad51 in UVA-irradiated HDF-α cells were determined by western blot assay using specific antibodies. (**F**) Relative protein expressions were quantified by densitometry and shown in the histogram. Data are expressed as the mean from three experiments. * *p* ≤ 0.05, *** *p* ≤ 0.001 indicate a significant difference.

UV exposure may elicit cell killing. Flow cytometry analysis revealed that the apparent increase by UV irradiation in the percentage of late apoptotic (Annexin V-PI double positive) and necrotic cells (PI positive), compared with those of un-irradiated HDF-α cells (*p* ≤ 0.05; *p* ≤ 0.05) (Figure 6A,B). Treatment with CGA (3 and 30 μM) remarkably decreased the proportions of these two types of cells. For an example, the percentage of late apoptosis cells was decreased from 18.4% at UVA-irradiated group to the 1.8% at 30 μM of CGA-treated group in HDF-α cells (*p* ≤ 0.05). Accordingly, the expression of cleaved-PARP, the key indicator for cell apoptosis, was notably increased after UVA-irradiation, which was decreased in a dose-dependent manner by treatment with CGA in HDF-α cells (Figure 6E,F).

### 2.6. CGA Inhibits the UVA-Induced ROS Production in HDFs

To understand further how CGA inhibited UV-irradiation induced apoptosis, we measured ROS production. As shown in Figure 6C,D, UVA irradiation for 60 min led to a marked increase in the levels of ROS compared with the control group (*p* < 0.01). However, combined treatment with CGA (30 μM) significantly decreased the accumulation of cellular ROS in UVA-irradiated HDF-α cells (*p* < 0.05). The level of γ-H_2_AX, the most sensitive marker of double strand breaks (DSBs), was also strikingly increased after UVA irradiation (Figure 6E). However, combined CGA with UVA irradiation obviously suppressed the γ-H_2_AX expression in HDF-α cells. The surrogate biomarker for DNA repair, Rad51, which was upregulated after UVA exposure and further increase after CGA treatment (Figure 6E).

## 3. Discussion

Our study, for the first time, demonstrated that CGA treatment increased the collagen biosynthesis and secretion in HDFs, especially in type 1 collagen through up-regulating Col1 mRNA and protein levels. Analysis for MMP1 and MMP3, of which mRNA levels were highly expressed than MMP9 in HDF-α cells, showed that CGA did not affect the MMP1 and MMP3 mRNA expressions in normal HDF-α cells (Supplementary Figure S1A,B). These data suggested that CGA mainly promoted the Col1 biosynthesis rather than suppressed its degradation in non-stressed HDFs.

UV radiation in sunlight is considered as the most prominent physical factor responsible for extrinsic skin aging. Chronic UVA radiation result in photosensitivity, photo-aging and the development of malignant skin tumor [25]. More importantly, consistent with previous reports [26], we found that UVA-irradiation significantly reduced the collagen production by down-regulating Col1, Col3 and Col5 gene levels, and stimulated collagen degradation by up-regulating the MMP1 and MMP3 mRNA levels. Combination with CGA remarkably reversed the Col1 and MMP3 expressions in UVA-irradiated HDF-α cells, resulting in improving the skin photoaging by enhancement of Col1 production and inhibition its degradation. Fibronectin is another vital component of the skin, which is deposited discretely along the dermal-epidermal junction. CGA attenuated the decrease of fibronectin after exposed to UVA, suggesting that CGA promotes the synthesis of other ECM products.

Many pro-fibrogenic growth factors and cytokines like TGF-β, IL-4, and IL-13 may bind to cell membrane receptors of fibroblasts and mediate Col1 biosynthesis. TGF-β is a major activator among them, which induces Smad 2/3 form a heterodimer complex with Smad 4, then this tertiary complex translocated into the cell nucleus to activate the procollagen promoter [27]. UVA irradiation can reduce the synthesis of TGF-β I and TGF-β II receptors to inhibit the TGF-β/smad pathway [23]. As expected, UVA irradiation inhibited the activation of Smad 2/3 signaling molecules in HDFs. CGA considerably increased the phosphorylated Smad 2/3 expressions in UVA-irradiated HDFs. Additionally, even though, the ERK pathway is an important MAPK signaling pathway that was established to involve in the activation of Col1 transcription [24], our study demonstrated that p-ERK1/2 was almost unchanged in UVA radiation alone or combined with CGA in HDFs. These results suggested that CGA promoted Col1 expression by stimulating TGF-β-Smad2/3 signaling pathway in UVA-irradiated HDFs.

It has been established that UV radiation cause significant oxidative stress through reactive oxygen species (ROS) produced in the epidermis or dermis. The accumulation of ROS increases MMP activity, resulting in destruction of extracellular matrix (ECM) via collagen degradation [28], but also evokes DNA damage and apoptosis of cells. Thus, natural phytochemicals, especially antioxidants are steadily increasing in cosmetics to protect against solar UV-induced oxidative damage to human skin. For example, curcumin exerted inhibitory effects on the UVB or UVA-induced ROS productions, and protected against UVA-mediated apoptosis by up-regulation of BCL2 protein in HDFs [29,30]. In our study, CGA significantly reduced γ-H_2_AX levels and further promoted Rad51 expressions in UVA-irradiated HDFs, suggesting that the improvement of the DNA DSBs damage and repair functions was concurrent with the decrease of cellular ROS productions after treatment with CGA, resulting in inhibition of apoptosis and necrotic cells in UVA-irradiated HDFs.

While this finding that CGA attenuated apoptosis or necrosis in UVA irradiated HDF-α cells seems counterintuitive to its role as anti-cancer therapeutics demonstrated in phase 1 study in solid tumors in China (http://www.chinadrugtrials.org.cn (accessed on 31 Jan 2022), number CTR20160113, and the USA clinical trial identifier http://clinicaltrials.gov (accessed on 31 Jan 2022), NCT02728349), it is consistent that CGA is not a direct apoptosis inducer by itself, nor does it inhibit proliferation in HDF-α cells as shown in this study. It is possible that UVA irradiation triggers different signal pathways in HDF-α cells than other cancer cells. Through regulating immune response, such as repolarizing M2 to M1 in glioblastoma, it elicits anti-tumor activity [15]. Interestingly, in the context of photoaging, for patients with glioblastoma during the phase 1 trial mentioned above, we had observed that the skin of some patients became whitening and brightening, mainly on the facial skin. The analysis for Col1 contents in the serum of CGA-treated GBM patients revealed that Col1 content was gradually increased after CGA therapy (Supplementary Figure S2), like what we discovered for Col1 increased by CGA in HDF models. It is not immediately clear such increase of Col1 in patients with glioblastoma treated with CGA, is a direct or the feedback protective effect. However, the impact of Col1 production on tumor angiogenesis and brain tumor progression needs to be elucidated in the further study.

**Figure 7:**
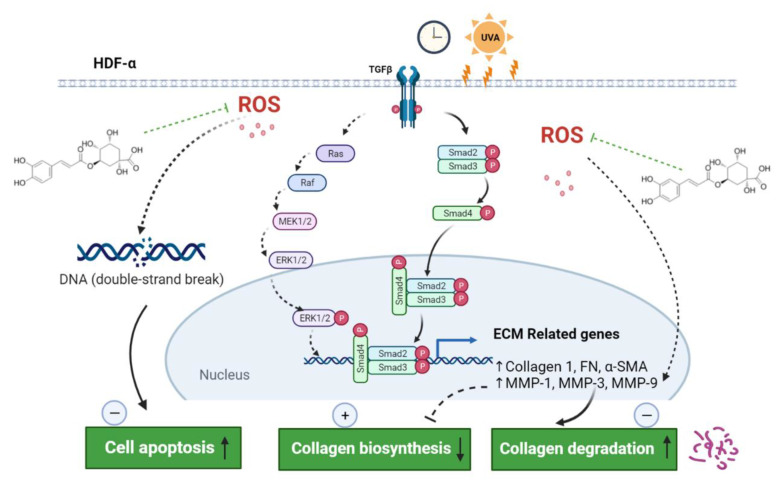
Schematic representation of the photoprotection of CGA on HDF-α cells. CGA promoted the collagen 1 synthesis through TGF-β-Smad2/3 signaling, and inhibited the collagen degradation by downregulating the MMP-1 and MMP-3. In addition, CGA reduced the accumulation of UVA-induced ROS, attenuated the DNA damage and promoted cell repair, resulting in inhibition the cell apoptosis. Green dotted T bars, potential inhibition; straight arrows, activation; dotted arrows, potential activation. ECM, extracellular matrix; MMP, matrix metalloproteinase; ROS, reactive oxygen species.

In conclusion, CGA promoted the Col1 synthesis under un-irradiated HDFs. Furthermore, CGA protected the HDF-α cells from UVA-induced photoaging by suppressing collagen degradation and enhancing collagen synthesis. In addition, CGA reduced the accumulation of UVA-induced ROS, attenuated the DNA damage and promoted cell repair. The underlying mechanism of photoprotection by CGA against UVA-induced photoaging was shown in Figure 7. Taken together, these findings support further investigation of CGA to be an effective therapeutic agent to prevent or treat skin photoaging and chronological aging.

## 4. Materials and Methods

### 4.1. Cells Culture

Human embryonic skin fibroblasts CCC-ESF-1 cells were supplied by the Cell Resource Center of Institute of Basic Medical Sciences, Chinese Academy of Medical Sciences (Peking Union Medical College, China). Primary human dermal fibroblasts-Adult (HDF-α) cells were purchased from ScienCell research libraries (San Diego, CA, USA). CCC-ESF-1 cell was cultured in Dulbecco’s modified Eagle’s medium (DMEM, Gibco^®^, Life Technologies^TM^, Carlsbad, CA, USA), supplemented with 10% fetal bovine serum (FBS) (Gibco, ThermoFisher Scientific, Waltham, MA, USA) and 100 units/mL penicillin, and 100 units/mL streptomycin. HDF-α cell was incubated in Fibroblast medium (FM, ScienCell Research Laboratories, Inc., San Diego, CA, USA, Cat. #2301) with 2% FBS, fibroblast growth supplement (FGS, ScienCell Research Laboratories, Inc., San Diego, CA, USA, Cat. #2352) and penicillin/streptomycin solution. These cells were incubated in a humidified atmosphere of 5% CO_2_ at 37 °C.

### 4.2. UVA Irradiation

HDF-α cells were seeded at a density of 2.5 × 10^5^/well of 6-well culture plates. After 24 h, the culture medium was replaced by PBS and exposed to UVA (12 J/cm^2^) for 60 min. The UVA source used in the experiment was a UV phototherapy instrument (PURI Materials, Shenzhen, China) with a peak emission at 365 nm. After UVA exposure, the cells were washed with PBS, then were incubated with DMSO (0.1%, control) or CGA (3, 30 μM) for 24 h to further experiments.

### 4.3. Antibodies and Chemicals

Antibodies against collagen 1 and fibronectin were obtained from abcam. MMP3, cleaved-PARP, Rad51, γ-H_2_AX, p-ERK1/2_Thr202/Tyr204_, ERK, p-smad2/3, smad2/3, β-actin and the corresponding HRP conjunction secondary antibodies were purchased from Cell Signaling Technologies (Danvers, MA, USA). MTT and DCFH-DA were purchased from Solarbio (Shanghai, China). Chlorogenic acid (CGA) was provided by Jiuzhang Biochemical Engineering Science and Technology Development Co., Ltd. (Chengdu, China), and dissolved in DMSO at the appropriate concentrations.

### 4.4. Cell Viability Assay

The MTT (3-(4,5-dimethylthiazol-2-yl)-2,5-diphenyl tetrazolium bromide) assay was adapted to measure the toxic effects of CGA on human dermal fibroblasts in vitro. HDF-α cells were seeded into 96-well plates. After overnight, the cells were treated with varying concentrations of CGA for 24, 48, and 72 h at 37 °C. Then, MTT (final concentration, 0.5 mg/mL) solution was incubated for 4 h. Subsequently, the medium was removed and the formazan crystals were solubilized in DMSO. The microplate reader (Biotek Instruments, Inc., Winooski, VT, USA) was used to determine absorbance at 570 nm.

### 4.5. Quantitative RT-PCR (qRT-PCR) Assay

Total RNA from HDF-α cells were extracted by the Easypure RNA kit (Tansgen Biotech, Beijing, China). cDNA was obtained using the TransScript One-Step gDNA Remove and cDNA Synthesis SuperMix kit (Tansgen, Beijing, China) following the manufacturer’s instructions. PCR amplifications were performed using a SYBR Green PCR Master Mix kit (Cat. QPK-201, Toyobo, Japan) and an ABI PRISM 7900 Sequence Detection system (Applied Biosystems, Foster City, CA, USA). The primers used for qPCR were as follows in Table 2. GAPDH was used as an internal control. The indicated gene expression was calculated according to the 2^−ΔΔCt^ method.

### 4.6. Western Blot Assays

CGA-treated HDF-α cells were lysed with ice-cold RIPA lysis buffer for 30 min. Equal protein amount from the supernatants were subjected by SDS-PAGE, proteins were transferred to PVDF membranes (Millipore, Burlington, MA, USA). After blocking with 5% BSA, the membranes were probed with specific primary antibodies, and further treated with the corresponding HRP-conjugated secondary antibodies. Proteins were then visualized by with Tanon Chemiluminescence Substrate, record by Image Quant LAS 4000 (GE Healthcare, Piscataway, NJ, USA) and analyzed with Image J software.

### 4.7. Soluble Collagen Detection

The HDF-α cells were plated in 24-well plates at a density of 1 × 10^5^ cells/well. Cells were treated with or without different concentrations of CGA. After 48 h, the supernatants were collected and total soluble collagen were quantified using the SirCol collagen assay (Biocolor, Belfast, Ireland). For these experiments, 100 μL of supernatant taken from treated cells were incubated with 1 mL of Sirius red dye, an anionic dye that reacts specifically with basic side-chain groups of collagens under assay conditions followed by incubation under gentle rotation for 30 min at room temperature. After centrifugation at 12,000 rpm for 10 min, the 750 μL of ice-cold acid-salt wash reagent was added to remove unbound dye. Subsequently, collagen-bound dye was redissolved with 250 μL of alkali reagent, and the absorbance was measured at 555 nm (Biotek Instruments, Inc., USA) according to manufacturer’s instructions. A calibration standard of type I collagen, provided with the assay, was used to obtain the standard curve.

### 4.8. BioMAP Profiling Analysis

Eight BioMAP systems were used in the present study to rapidly permit the characterization of CGA function. In the BioMAP HDF3CGF system, primary human neonatal foreskin fibroblasts (HDF-n) were plated in low serum conditions 24 h before stimulation with cytokines in Table 1 After incubation with indicated doses of CGA for 72 h, a set of biomarkers (protein) readouts described in Table 1 were measured by ELISA. The method used in this study was essentially the same as previously described [31].

### 4.9. Apoptosis Assay

The HDF-α cells were treated with CGA after UVA-irradiation (12 J/cm^2^). After incubation for 24 h, the HDF-α cells were harvested and resuspended in 100 μL of staining buffer. Then, 5 μL of Annexin V-APC and 10 μL of propidium iodide (PI) were added and incubated for 5 min in the dark. The early and late apoptosis cells and necrosis cells were analyzed using flow cytometry (BD FACSVerse^TM^, BD Biosciences, San Jose, CA, USA).

### 4.10. Cellular ROS Detection Assay

The HDF-α cells were plated in 6-well plates at a density of 3 × 10^5^ cells/well. Cells were treated with or without CGA (final concentrations of 3 and 30 μM) immediately after UVA irradiation (12 J/cm^2^). After incubation for 24 h, the HDF-α cells were collected and the intracellular ROS production was determined by Reactive Oxygen Species Assay Kit (Solarbio, CA1410, Shanghai, China) using DCFH-DA ROS probe according to the manufacturer’s instructions. The HDF-α cells were stained with 10 μM DCFH-DA working buffer for 30 min in 37 °C with 5% CO_2_. Then, cells were harvested and analyzed using BD FACSVerse^TM^ Flow cytometry.

### 4.11. Statistical Analysis

The data are expressed as the means ± standard deviation (S.D) using triplicate values. Comparisons used two-tailed Student’s *t*-test. Differences were considered significant at *p* ≤ 0.05.

## Figures and Tables

**Table 1:** The BioMAP HDF3CGF system utilized in the screening study. The cell types shown cultured and stimulated with the environmental factors in the presence of CGA for 72 h. The biomarker readouts listed were measured.

System	Cell Type	Environment	Readouts
HDF3CGF 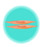	Dermal fibroblasts	IL-1β + TNF-α + IFNγ+ bFGF + EGF + PDGF-BB	MCP-1, VCAM-1, ICAM-1, Collagen Ⅰ, Collagen Ⅲ, IP-10, I-TAC, IL-8, MIG, EGFR, M-CSF, MMP-1, PAI-1, Proliferation-72 h, SRB, TIMP-1, TIMP-2

**Table 2:** The primer sequences in qRT-PCR experiments.

Target	Primer Sequence (5′-3′)
hCol1A1-F	GTGCGATGACGTGATCTGTGA
hCol1A1-R	CGGTGGTTTCTTGGTCGGT
hCol1A2-F	GGCCCTCAAGGTTTCCAAGG
hCol1A2-R	CACCCTGTGGTCCAACAACTC
hCol3A1-F	TTGAAGGAGGATGTTCCCATCT
hCol3A1-R	ACAGACACATATTTGGCATGGTT
hCol5A1-F	TACCCTGCGTCTGCATTTCC
hCol5A1-R	GCTCGTTGTAGATGGAGACCA
hMMP1-F	AAAATTACACGCCAGATTTGCC
hMMP1-R	GGTGTGACATTACTCCAGAGTTG
hMMP3-F	CTGGACTCCGACACTCTGGA
hMMP3-R	CAGGAAAGGTTCTGAAGTGACC
hMMP9-F	TGTACCGCTATGGTTACACTCG
hMMP9-R	GGCAGGGACAGTTGCTTCT
hGAPDH-F	GGAGCGAGATCCCTCCAAAAT
hGAPDH-R	GGCTGTTGTCATACTTCTCATGG

## Data Availability

Data is contained within the article.

## Supplementary Materials

The following are available online at https://www.mdpi.com/article/10.3390/ijms23136941/s1.

## Abbreviations

CGA	Chlorogenic acid
HDFs	Human dermal fibroblasts
Col	Collagen
DSBs	Double strand breaks
MMPs	Matrix metalloproteinases
ECM	Extracellular matrix
UVA	Ultraviolet A
ROS	Reactive oxygen species
TGF-β	Transforming growth factor-β
