# Supplementary material for: Chlorogenic Acid Prevents UVA-Induced Skin Photoaging through Regulating Collagen Metabolism and Apoptosis in Human Dermal Fibroblasts
Source: Int J Mol Sci. 2022 Jun 22;23(13):6941. doi: 10.3390/ijms23136941 (PMC9266774; doi:10.3390/ijms23136941)
Supplement: Supplementary file 1 [file ijms-23-06941-s001.zip › ijms-1765848-supplementary.pdf]

Chlorogenic acid prevents UVA-induced skin photoaging through regulating collagen metabolism and apoptosis in human dermal fibroblasts

Nina Xue, Ying Liu, Jing Jin, Ming Ji, Xiaoguang Chen*

State Key Laboratory of Bioactive Substances and Functions of Natural Medicines, Institute of Materia Medica, Chinese Academy of Medical Sciences, Peking Union Medical College, Beijing 100050, China.

* Correspondence and requests for materials should be addressed to

X.C. (email: chxg@imm.ac.cn)

Method

Measurement of type 1 collagen in human GBM patients by ELISA. The serum type 1 collagen concentrations of human recurrent high-grade glioblastoma patients from the clinical I trail study (CTR20160113, NCT02728349) were measured according to the human collagen ELISA kit protocol (SEA571Hu; Cloud-Clone Corp). The human samples used in this experiment were as follows: Day 0, Day 16 and Day28 after administration with CGA. The type 1 collagen content change was calculated with the famu:100%×($Conc_{day16/28}$-$Conc_{day0}$)/ $Conc_{day16/28}$. This study was conducted in accordance with the Good Clinical Practice guidelines, the Declaration of Helsinki, and the corresponding Regulations issued by National Medical Products Administration (NMPA). The protocol was approved by the institutional review board of Beijing Shijitan Hospital.

Results

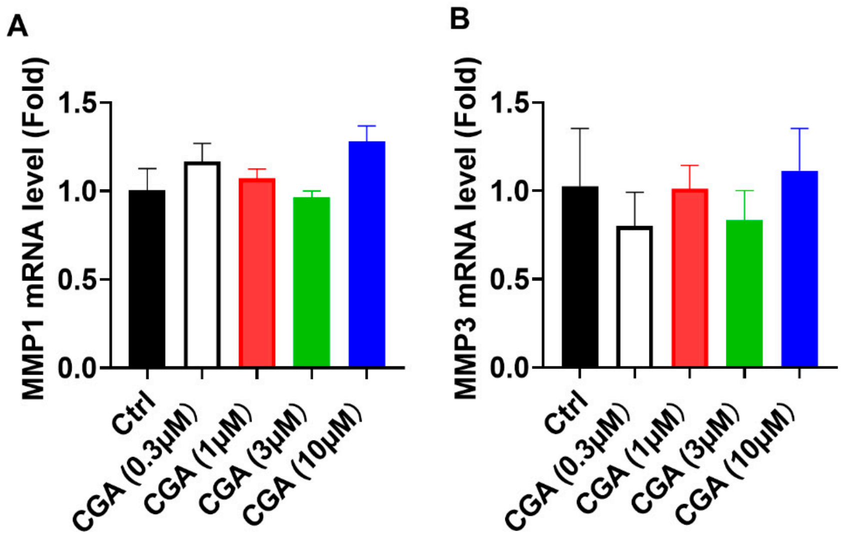

Figure S1. The effect of CGA on MMPs expression in human dermal fibroblasts. The MMP1 (**A**) and MMP3 (**B**) mRNA levels were measured by qRT-PCR assay after treatment with CGA for 48 h in HDF-α cells. The values are expressed as the mean ± SD from three experiments.

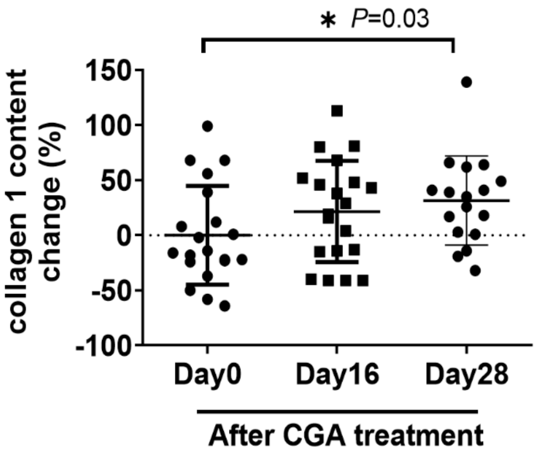

Figure S2. The effect of CGA on collagen I productions in GBM patients of phase 1 clinical. The changes of collagen I contents in the serum of GBM patients after administration of CGA were detected by ELISA assay. The values are expressed as the mean ± SD. * $p < 0.05$ indicate a significant difference.
